# Sulphuric acid-mediated weathering on Taiwan buffers geological atmospheric carbon sinks

**DOI:** 10.1038/s41598-019-39272-5

**Published:** 2019-02-27

**Authors:** T. M. Blattmann, S.-L. Wang, M. Lupker, L. Märki, N. Haghipour, L. Wacker, L.-H. Chung, S. M. Bernasconi, M. Plötze, T. I. Eglinton

**Affiliations:** 1 Geological Institute, ETH Zurich, Sonneggstrasse 5, 8092 Zurich, Switzerland; 2 Department of Geosciences, National Taiwan University, No. 1, Sec. 4, Roosevelt Rd, Taipei, 10617 Taiwan; 3 Department of Oceanography, National Sun Yat-sen University, 70 Lienhai Rd., Kaohsiung, 80424 Taiwan; 4 Ion Beam Physics, ETH Zurich, Otto-Stern-Weg 5, 8093 Zurich, Switzerland; 5 National Museum of Natural Science, No. 1, Guanqian Rd., Taichung, 40453 Taiwan; 6 Institute for Geotechnical Engineering, ETH Zurich, Stefano-Franscini-Platz 3, 8093 Zurich, Switzerland

## Abstract

The chemical composition of the Gaoping River in Taiwan reflects the weathering of both silicate and carbonate rocks found in its metasedimentary catchment. Major dissolved ion chemistry and radiocarbon signatures of dissolved inorganic carbon (DIC) reveal the importance of pyrite-derived sulphuric acid weathering on silicates and carbonates. Two-thirds of the dissolved load of the Gaoping River derives from sulphuric acid-mediated weathering of rocks within its catchment. This is reflected in the lowest reported signatures DI^14^C for a small mountainous river (43 to 71 percent modern carbon), with rock-derived carbonate constituting a ^14^C-free DIC source. Using an inverse modelling approach integrating riverine major dissolved ion chemistry and DI^14^C, we provide quantitative constraints of mineral weathering pathways and calculate atmospheric CO_2_ fluxes resulting from the erosion of the Taiwan orogeny over geological timescales. The results reveal that weathering on Taiwan releases 0.31 ± 0.12 MtC/yr, which is offset by burial of terrestrial biospheric organic carbon in offshore sediments. The latter tips the balance with respect to the total CO_2_ budget of Taiwan such that the overall system acts as a net sink, with 0.24 ± 0.13 MtC/yr of atmospheric CO_2_ consumed over geological timescales.

## Introduction

Taiwan is one of the most rapidly uplifting orogens, with erosion rates in the order of 3–6 mm/yr continuously exposing fresh minerals for chemical weathering^1,2^. Together with volcanic activity, metamorphic degassing, and the organic carbon cycle, chemical weathering of minerals exerts a key control on atmospheric chemistry over geologic timescales^3^. Orogenies sustain high rates of physical erosion and are classically invoked as major CO_2_ sinks due to the weathering of silicates by carbonic acid^4,5^. While carbonic acid as a weathering agent is widely considered the most important, recent work has highlighted that sulphuric acid weathering of carbonates plays an important role in catchments that contain abundant pyrite^2,6–9^ and acts as a major source of CO_2_ to the atmosphere over geological timescales^10–12^. Pyrite oxidises to sulphuric acid giving rise to river dissolved sulphate^6,8^ following the weathering pathway^10^:

1 $${{\rm{4FeS}}}_{{\rm{2}}}+{{\rm{15O}}}_{{\rm{2}}}+{{\rm{14H}}}_{{\rm{2}}}{\rm{O}}\to {\rm{4Fe}}{({\rm{OH}})}_{{\rm{3}}}+{{\rm{8H}}}_{{\rm{2}}}{{\rm{SO}}}_{{\rm{4}}}$$

Together with carbonic acid and sulphuric acid stemming from weathering reaction (1), silicate and carbonate mineral weathering proceeds as follows

2 $${{\rm{CaSiO}}}_{{\rm{3}}}+{{\rm{2H}}}_{{\rm{2}}}{{\rm{CO}}}_{{\rm{3}}}+{{\rm{H}}}_{{\rm{2}}}{\rm{O}}\to {{\rm{Ca}}}^{{\rm{2}}+}+{{\rm{H}}}_{{\rm{4}}}{{\rm{SiO}}}_{{\rm{4}}}+{{{\rm{2HCO}}}_{{\rm{3}}}}^{-}$$

3 $${{\rm{CaSiO}}}_{{\rm{3}}}+{{\rm{H}}}_{{\rm{2}}}{{\rm{SO}}}_{{\rm{4}}}+{{\rm{H}}}_{{\rm{2}}}{\rm{O}}\to {{\rm{Ca}}}^{{\rm{2}}+}+{{\rm{H}}}_{{\rm{4}}}{{\rm{SiO}}}_{{\rm{4}}}+{{{\rm{SO}}}_{{\rm{4}}}}^{{\rm{2}}-}$$

4 $${{\rm{CaCO}}}_{{\rm{3}}}+{{\rm{H}}}_{{\rm{2}}}{{\rm{CO}}}_{{\rm{3}}}\to {{\rm{Ca}}}^{{\rm{2}}+}+{{{\rm{2HCO}}}_{{\rm{3}}}}^{-}$$

5 $${{\rm{C}}{\rm{a}}{\rm{C}}{\rm{O}}}_{3}+\frac{1}{2}{{\rm{H}}}_{2}{{\rm{S}}{\rm{O}}}_{4}\to {{\rm{C}}{\rm{a}}}^{2+}+{{{\rm{H}}{\rm{C}}{\rm{O}}}_{3}}^{-}+\frac{1}{2}{{{\rm{S}}{\rm{O}}}_{4}}^{2-}$$

The trails of evidence of these weathering reaction pathways (2–5) lead to unique signatures in the dissolved ion load (see Table 1). The theoretical radiocarbon isotopic composition of DIC (hereafter DI^14^C) arising from these pathways are characteristic for different weathering mechanisms. In reaction (2), carbon is sourced primarily from the atmosphere, which exhibits a modern signature, expressed as 100% modern carbon (pMC). Reaction (3) does not involve carbon and only adds sulphate to the dissolved river load. In reaction pathway (4), 50% of bicarbonate carbon is sourced from the atmosphere and the other half from the radiocarbon-dead lithosphere, hence characterised by a pMC = 50 “fingerprint”, while in the case of (5), bicarbonate is entirely derived from the radiocarbon-dead lithospheric source (i.e., pMC = 0). Radiocarbon has considerable advantages over stable carbon isotopic compositions of DIC by incorporating a correction for fractionation and exhibiting lower end-member uncertainty^13,14^. Additionally, the weathering of silicates and carbonates release characteristic assemblages of major cations (calcium, magnesium, and sodium) to the river dissolved load that are further indicative of the mineral species undergoing chemical weathering^5^.

**Table 1:** Theoretical stoichiometries of weathering reactions and their DI^14^C fingerprints.

Mineral type	Weathering Pathway	HCO_3_	SO_4_	pMC	$\frac{{\bf{Na}}}{{\boldsymbol{(}}{\bf{H}}{\bf{C}}{{\bf{O}}}_{{\bf{3}}}{\boldsymbol{+}}{\bf{S}}{{\bf{O}}}_{{\bf{4}}}{\boldsymbol{)}}}$	$\frac{{\bf{Ca}}}{{\boldsymbol{(}}{\bf{H}}{\bf{C}}{{\bf{O}}}_{{\bf{3}}}{\boldsymbol{+}}{\bf{S}}{{\bf{O}}}_{{\bf{4}}}{\boldsymbol{)}}}$	$\frac{{\bf{Mg}}}{{\boldsymbol{(}}{\bf{H}}{\bf{C}}{{\bf{O}}}_{{\bf{3}}}{\boldsymbol{+}}{\bf{S}}{{\bf{O}}}_{{\bf{4}}}{\boldsymbol{)}}}$
Silicate	Carbonic acid	2	0	100	0.42	0.15	0.11
	Sulphuric acid	0	1	Undef.	0.85	0.30	0.21
Carbonate	Carbonic acid	2	0	50	0.006	0.33	0.17
	Sulphuric acid	1	0.5	0	0.007	0.44	0.22

Bicarbonate and sulphate concentrations normalised to one silicate or carbonate mineral unit (Ca, Mg, 2Na, 2K)SiO_3_ or (Ca, Mg, 2Na, 2K)CO_3_ following Eqs (2–5). The theoretical average major anion molar concentration-normalised sodium, calcium, and magnesium concentrations are given for the different minerals types and their weathering pathways.

Globally, the reaction pathway of silicates and carbonates dictates the net effect of weathering on atmospheric CO_2_^3,4^, with the dissolution of carbonates by sulphuric acid acting as a CO_2_ source over geological time scales^10^. Using a novel approach, we quantitatively disentangle the inputs of silicate and carbonate weathering via carbonic and sulphuric acid dissolution by determining DI^14^C and dissolved ion compositions within the Gaoping River catchment of Taiwan, leading to new quantitative estimates on the effect of the Taiwan orogeny on atmospheric chemistry.

## Study Area and Methods

The Gaoping River covers a length of 170 km and drains 3257 km^2^ in southwest Taiwan and is the island’s second largest river as measured by sediment discharge^6,15^. Nearly half of its catchment is situated above 1000 m elevation, reaching a maximum of 3997 masl^15^. Within the catchment, (meta) sedimentary rocks ranging from shales to conglomerates of ages spanning from the Mesozoic to Pleistocene are exposed (see appendix for details and references). Due to the monsoonal climate, >90% of river discharge takes place in the flood season (focused in June to October)^15^.

**Figure 1:**
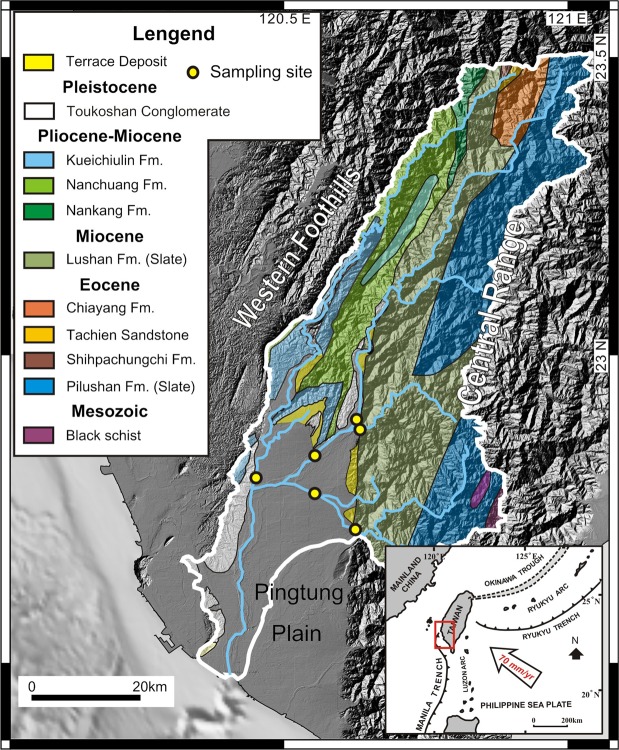
Gaoping River catchment. Geological and hydrological overview with sampling locations. Redrawn after geologic map of Taiwan^44^, which is openly accessible (https://data.gov.tw/dataset/11004). Topographic features were added from open access digital elevation data (https://data.gov.tw/dataset/35430) and shaded with QGIS (QGIS Development Team (2018). QGIS Geographic Information System. Open Source Geospatial Foundation Project. www.qgis.org). The Gaoping River catchment was outlined from open access data from the Taiwanese government (https://www.wra07.gov.tw/12594/12595/12602/12605/70918/) and the final figure was generated with CorelDRAW (www.coreldraw.com).

Surface water samples were collected from the Gaoping River catchment during the dry season in February 2017 and 2018 as well as during the wet season in June and October 2017 (Fig. 1). Filtered water samples were enclosed in 12 ml glass exetainer vials without head-space and pre-poisoned with 12 µl of dried HgCl_2_ saturated solution. One and six millilitre aliquots were purged with He, acidified with 150 µl 85% H_3_PO_4_, and measured for their DI^13^C and DI^14^C isotope compositions, respectively, using online CO_2_ sparging-mass spectrometry setups described elsewhere^16,17^. Radiocarbon values are reported in units pMC^18^. Concentrations of major cations and anions were measured by ion chromatography with details reported in the appendix. Bicarbonate concentrations where calculated by charge balance, following previous Taiwanese river studies^2,8,12,19^. Inputs from rainwater were removed by assuming all chloride is sourced from the atmosphere and by subtracting ions using ratios typical for rainwater (Ca/Na = 0.023; Mg/Na = 0.11; Cl/Na = 1.15; S/Na = 0.06; HCO_3_/Na = 0.004)^5,7^.

Using a constrained linear least-squares approach with the Matlab solver lsqlin, contributions from weathering reactions (2–5) are assessed from the mineral unit-normalised stoichiometries (see definition below), DI^14^C fingerprints, and ionic ratios characteristic for different weathering reactions listed in Table 1. Here we use α to denote the relative contributions stemming from the carbonic acid weathering of silicates (α_Silicate,H2CO3_) and carbonates (α_Carbonate,H2CO3_) and sulphuric acid weathering of silicates (α_Silicate,H2SO4_) and carbonates (α_Carbonate,H2SO4_). The model output is bound by three equality constraints: (1) the sum of the weathering contributions α_i_ for the mineral-normalised ion concentrations for the four reactions equals 100%, (2) measured and modelled mineral unit-normalised SO_4_ must be equal, and (3) the measured and modelled DI^14^C must be equal. Additional constraints are included to find the best least-squares fit between modelled and measured dissolved major ion composition based on ratios between calcium, magnesium, and sodium and the sum of sulphate and bicarbonate, which are indicative of different weathering pathways from silicates and carbonates (see Table 1 and appendix for equations). Here, we define a “mineral unit” as (Ca, Mg, 2Na, 2 K)SiO_3_ for silicates and (Ca,Mg,2Na,2 K)CO_3_ for carbonates. Within each silicate and carbonate mineral unit, calcium is interchangeable with charge balance equivalent amounts of magnesium, sodium, and potassium. For silicates, the relative molar abundances of these cations are typically 0.35 ± 0.25 for Ca/Na and 0.25 ± 0.2 for Mg/Na. For carbonates, Ca/Na and Mg/Na are 60 ± 30 and 30 ± 15, respectively^5,7^, with the uncertainties taken to represent 2-σ uncertainty in the model. Potassium is also present in carbonate mineral lattices in minor quantities (Ca/K ≈ 250–350)^20^, besides its presence in micas and potassic feldspars. Based on these ratios and the ideal mineral unit definition introduced here, the average formula for carbonates and for silicates is Ca_0.66_Mg_0.33_Na_0.011_K_0.0055_CO_3_ and Ca_0.30_Mg_0.21_Na_0.85_K_0.14_SiO_3_, respectively. Model output uncertainty was quantified by applying a Monte Carlo approach (10,000 iterations) propagating analytical uncertainties from measured ionic ratios (5% relative 1-σ uncertainty assigned), measured DI^14^C uncertainty (≈1% 1-σ uncertainty based on analytical uncertainty), uncertainty in the cation composition of the weathered minerals, and uncertainty in the radiocarbon signature of the carbonic acid weathering agent. In the case of the latter, an end member signature of 100 ± 5 (1-σ uncertainty) pMC was assigned, with the uncertainty stemming from possible contributions of respired soil organic carbon^21,22^ or degraded kerogen, which attenuate the atmospheric carbon signature with the addition of bomb and aged carbon isotopic signatures. Used equations are reported in the appendix.

## Results and Discussion

Over the sampled time interval, pH averaged 7.9 ± 0.2 (n = 13) and bicarbonate represented the major anion (2.3 ± 0.6 mmol/l) followed by sulphate (0.9 ± 0.4 mmol/l) and chloride (0.2 ± 0.3 mmol/l) (supplemental Table 1). For the cations, calcium was the most abundant (1.4 ± 0.4 mmol/l n = 13) followed by magnesium and sodium (both 0.5 ± 0.2 mmol/l), and potassium (0.2 ± 0.2 mmol/l). Carbon isotopic compositions were centred at −5.3 ± 1.3‰ for DI^13^C (n = 13), while DI^14^C values ranged from 43 to 68 pMC (average 54 ± 8 pMC n = 20) (supplemental Table 1). One sample contained 0.02 mmol/l of ammonium that was presumably sourced from anthropogenic inputs and was also characterized by the highest nitrate concentrations suggesting that nitrification induced mineral weathering was ongoing^23,24^. As this additional weathering pathway could not be deconvolved using the approaches devised here, this sample (2018FebAR) was not considered further in this study.

**Figure 2:**
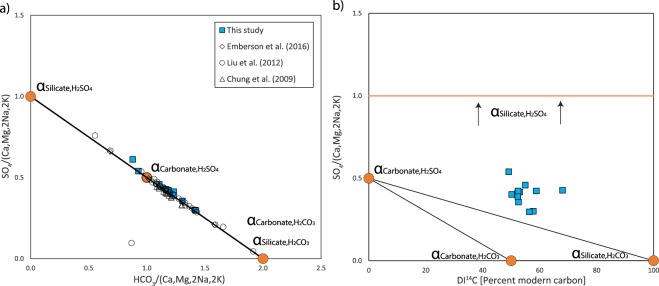
Mineral weathering end-member mixing. (**a**) Mineral unit-normalised sulphate versus bicarbonate abundance. The Gaoping River data collected over multiple years in relation to the quaternary end-member mixing line. Literature data points where ionic compositions reflect incursion of seawater plot away from the quaternary mixing line. One data point from this study (datapoint with highest mineral unit normalised sulphate) deviated from the mixing line due to high ammonium amounts that are attributed to anthropogenic inputs and therefore wasn’t considered further in this study. Removing these anomalies, the data from this study correlates with R^2^ = 0.99, and for all studies with R^2^ = 0.99. (**b**) Mineral unit-normalised sulphate versus pMC of DIC. The three mineral weathering reactions producing bicarbonate span a ternary mixing triangle. All of the data lie outside of this mixing ternary due to the addition of sulphate from sulphuric acid weathering of silicates. The latter process, which leaves DI^14^C on the x-axis unaffected, adds sulphate until a theoretical maximum of one mole per weathered mineral unit, representing 100% silicate weathering by sulphuric acid.

Taiwanese bedrock primarily consists of (metamorphosed) sandstones, siltstones, and claystones, up to a metamorphic grade of amphibolite facies with no reports of evaporitic deposits occurring on the island^25^ (see also Supplemental Table 2). In agreement with these geological observations, sulphur isotopes in sulphate^24,26^ demonstrate that pyrite oxidation is the predominant source of dissolved sulphate in Taiwanese rivers^6,24,26^. Following the theoretical outline provided in the introduction, rainwater-corrected river data from the Gaoping River are plotted in Fig. 2a showing the mixing of the four end members, which adhere to theoretical expectations in the case of quaternary mixing of solute derived from silicate and carbonate weathering via sulphuric and carbonic acid in the absence of evaporite contributions (Fig. 2a). The relatively high concentrations of chloride ions are similar to those of New Zealand rivers within catchments that receive high amounts of precipitation, which is also attributed to cyclic salt input^27^. Based on the measured DI^13^C values, soil organic matter and kerogen degradation are considered to contribute subordinately to the DIC pool. Similarly low contributions are also inferred for other Taiwanese rivers where most DI^13^C values suggest a predominantly carbonate source for riverine DIC^12^, supporting the assumption in our model that DIC is exclusively carbonate mineral-derived. Figure 2b shows the effect of ternary mixing between reactions (2), (4), and (5). Excess sulphate is introduced into the system via reaction (3), which has no effect on the DI^14^C composition, yet causes the data points to lie outside the boundaries for ternary mixing. By applying the equations outlined in the methods and using the stoichiometric constraints summarised in Table 1 on the data reported in Table S1, the relative contributions stemming from mineral weathering pathways are calculated. These calculations deconvolve the excess sulphate contributions in order to allow quantification of the weathering contributions controlling DI^14^C under the constraint of ionic composition adhering to the quaternary mixing line. The weathering apportionment did not reveal any clear patterns as a function of sampling season or location. Averaged over the catchment and the seasons, and based on molar mineral unit-normalised weathering, α_Silicate,H2CO3_ and α_Carbonate,H2CO3_ averaged 34 ± 5% and 0.5% (−0.5/+2%) respectively, while α_Silicate,H2SO4_ and α_Carbonate,H2SO4_ averaged 14 ± 9% and 53 ± 8%, respectively (see also Supplemental Fig. S1–S13). Total weathered silicates (α_Silicate,H2CO3_ + α_Silicate,H2SO4_) contribute 46 ± 8% and carbonates (α_Carbonate,H2CO3_ + α_Carbonate,H2SO4_) contribute 54 ± 8% to the total dissolved ion load, which is broadly consistent with previous observations of major cation chemistry from the Gaoping River^2,19,28^ (see Appendix Fig. S16). Integrated over the Gaoping River catchment, the weathering effect of carbonic acid (α_Silicate,H2CO3_ + α_Carbonate,H2CO3_ = 33 ± 6%) and sulphuric acid (α_Silicate,H2SO4_ + α_Carbonate,H2SO4_ = 67 ± 6%) account for one-third and two-thirds of the total weathering, respectively. The results reveal that carbonates are almost exclusively weathered by sulphuric acid. This is most likely due to the limited abundance of rock carbonate (as siliciclastic metasedimentary units dominate in the catchment of the Gaoping River), a high supply of sulphuric acid, and the rapid reaction kinetics of sulphuric acid with carbonates. The excess sulphuric acid continues to weather silicates. The weathering patterns observed for the Gaoping catchment operate comparably for other Taiwanese catchments including the Liwu^8^, Taimaili^12^, and Chenyoulan^12^ rivers. This is evidenced by their adherence to the quaternary mixing trend, encouraging the extrapolation of the results generated here to the entire Taiwan orogenic belt (see appendix).

**Figure 3:**
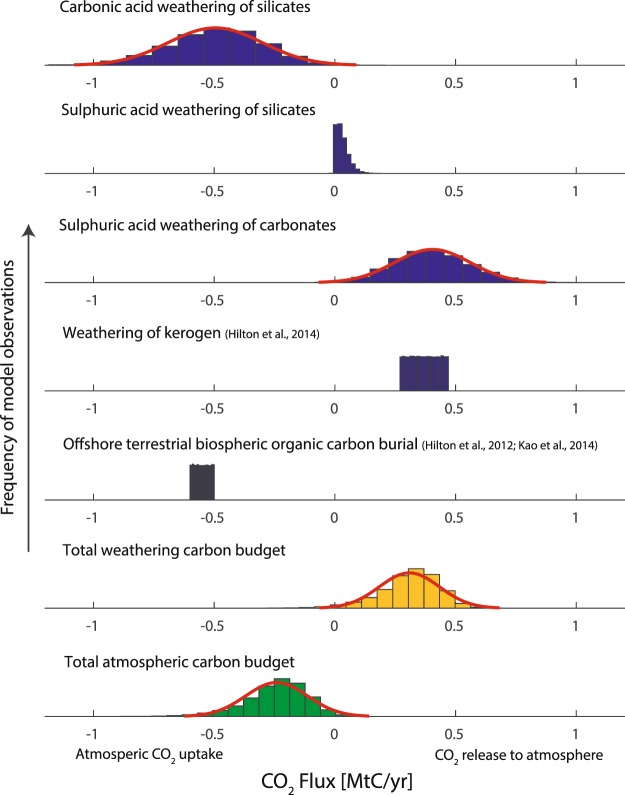
Fluxes of atmospheric CO_2_. Net total CO_2_ balance of Taiwan in MtC/yr with positive and negative values representing an atmospheric source and sink, respectively. The effects of CO_2_ drawdown and release by mineral weathering (this study) and organic geochemical cycles^29,32,33^ are compared.

In the case of Taiwan and major orogenic phases (e.g. Himalayan orogeny), meta-sediments are uplifted bearing the mineral ingredients to both sequester CO_2_ and also release it to the atmosphere^4,10,29^. Based on Taiwanese long-term discharge measurements (4.98 × 10^13^ l/yr^1^), the average mineral-unit equivalent normalised dissolved ion load (2.5 ± 0.9 mmol/l n = 63) observed for the Gaoping River catchment (this study^2,19,28^), and the calculated mineral weathering apportionment with propagated uncertainty, 0.50 ± 0.19 MtC/yr are removed from the atmosphere as a result of carbonic acid weathering of silicates following carbonate precipitation in the oceans. In contrast, 0.40 ± 0.16 MtC/yr are released long term as a result of sulphuric acid weathering of carbonates. While sulphuric acid weathering of silicates does not directly involve carbon, for each silicate mineral unit weathered, ≈0.45 mineral units of carbonate can be precipitated by combining the dissolved cations generated with bicarbonate, which then releases CO_2_ to the atmosphere. Based on the ideal mineral unit formula, 0.30 moles of calcium are released upon weathering of one silicate unit and 0.66 moles of calcium are needed for one carbonate unit. Over geological timescales, sulphuric acid weathering of silicates on Taiwan may thus result in the release of 0.09 ± 0.07 MtC/yr (see second panel in Fig. 2). The net release of CO_2_ to the atmosphere from chemical weathering of minerals as interpreted in this study is consistent with previous work incorporating considerations of alkalinity and DIC delivered to the ocean^30^. CO_2_ release is expected to occur on timescales longer than that of carbonate burial (10^5^-10^6^ years), yet shorter than pyrite burial (10^7^ years)^30^ (see Fig. S14 in appendix). In addition to chemical weathering of minerals, the weathering of kerogen (CO_2_ source), quantified by river dissolved rhenium, results in the release of 0.27–0.47 MtC/yr^29^. To counterbalance this, the burial of terrestrial biospheric carbon in adjacent ocean sediments^31,32^, quantified previously using radiocarbon as a tracer for modern sedimentary organic matter, removes 0.5–0.6 MtC/yr^32,33^. In terms of overall carbon balance, the long-term weathering effect of silicates, carbonates, and kerogen on land, together with offshore burial of terrestrial biospheric organic carbon, results in the removal of 0.24 ± 0.13 MtC/yr from the atmosphere. However, the total weathering effect on land acts as a net source of CO_2_ to the atmosphere, releasing 0.31 ± 0.12 MtC/yr (see Fig. 3). These quantities illustrate the fine balance between weathering mechanisms, and of the need to disentangle underlying processes for accurate assessment of the net effects of weathering budgets on atmospheric chemistry. The Taiwan orogeny shows that the weathering of metasedimentary catchments rich in pyrite and kerogen significantly influence the carbon budget of an orogenic entity.

The carbon budget presented here should be viewed conservatively, as two additional sources of CO_2_ to the atmosphere, authigenic silicate formation^34^ and metamorphic degassing^35^, are not considered. Considerable uncertainty lies in the former, as this process is limited by reactive silica availability in the oceans^34^. In the case of the latter process, the emission of CO_2_ and volatile hydrocarbons (e.g. CH_4_) further offset net carbon sequestration as a result of earth surface geochemical pathways and may tip the integrated surface and deep Earth geochemical cycles of an orogeny towards acting as a carbon source to the atmosphere^35,36^. Changes through time can be expected with erosional events^2^ as a result of major typhoons^37^ and mineral weathering pathways modified by fertiliser usage^23^. From the available data, the key variable compensating the release of CO_2_ to the atmosphere due to chemical weathering is the export and preservation efficiency of terrestrial biospheric organic carbon in offshore sediments – a particularly dynamic component that is responsive to tectonic^38^, climatic^31,39^ and anthropogenic^40^ influences.

The relative importance of sulphuric acid-mediated weathering depends on the presence and weathering of pyrite in exposed lithologies. The combined contributions of the Gaoping, Mackenzie (northern Canada), and Jialing (southern China) rivers deliver around 12% of sulphide-derived sulphate to the oceans, while their catchments encompass less than 2% of the Earth’s land surface^7^, highlighting the uneven distribution and localized occurrence of sulphuric acid-mediated weathering. In metasedimentary catchments such as those of the Gaoping River on Taiwan, weathering by sulphuric acid may contribute significantly to mineral dissolution. Here, it accounts for approximately two-thirds of total mineral dissolution, with carbonates almost entirely dissolved by sulphuric acid. The chemical weathering carbon budget of Taiwan supports the hypothesis that sulphuric acid mediated weathering in tandem with orogenic activity may well have affected atmospheric chemistry over geologic time^10^. Since the rise in atmospheric oxygen in the Precambrian, pyrite oxidation and sulphuric acid evolved therefrom has affected ocean chemistry, biologic activity, and atmospheric oxygen concentrations^41–43^. Mineral weathering by sulphuric acid has important implications for atmospheric CO_2_ inventories over geologic time^10,30^, with CO_2_ uptake and release governed by the fine balance between organic carbon burial in sediments and silicate, carbonate, and kerogen weathering controlled by the activity of oxygen, carbonic acid, and sulphuric acid. The total effect of orogenic activity on atmospheric chemistry remains a matter for discussion^36^, yet it is clear that the presence of sulphuric acid and its involvement in carbonate and silicate mineral weathering acts as a strong buffer on atmospheric CO_2_ uptake.

## Supplementary information

Supplementary online information (appendix)

Table S1
