# Supplementary material for: Sulphuric acid-mediated weathering on Taiwan buffers geological atmospheric carbon sinks
Source: Sci Rep. 2019 Feb 27;9:2945. doi: 10.1038/s41598-019-39272-5 (PMC6393438; doi:10.1038/s41598-019-39272-5)
Supplement: Supplementary file 1 — Supplementary online information (appendix) [file 41598_2019_39272_MOESM1_ESM.docx]

Supplementary information for the manuscript:

Sulphuric acid-mediated weathering on Taiwan buffers geological atmospheric carbon sinks

T. M. Blattmann^1*^, S.-L. Wang^2,3^, M. Lupker^1^, L. Märki^1^, N. Haghipour^1^, L. Wacker^4^, L.-H. Chung^5^, S. M. Bernasconi^1^, M. Plötze^6^, and T. I. Eglinton^1^

^1^Geological Institute, ETH Zurich, Sonneggstrasse 5, 8092 Zurich, Switzerland

^2^Department of Geosciences, National Taiwan University, No. 1, Sec. 4, Roosevelt Rd., Taipei 10617, Taiwan

^3^Department of Oceanography, National Sun Yat-sen University, 70 Lienhai Rd., Kaohsiung 80424, Taiwan

^4^Ion Beam Physics, ETH Zurich, Otto-Stern-Weg 5, 8093 Zurich, Switzerland

^5^National Museum of Natural Science, No. 1, Guanqian Rd., Taichung 40453, Taiwan

^6^Institute for Geotechnical Engineering, ETH Zurich, Stefano-Franscini-Platz 3, 8093 Zurich, Switzerland

*) thomas.blattmann@erdw.ethz.ch

For Table S1, please download excel file in supplementary online information.

**Catchment lithology**

Outcropping lithologies in the Gaoping River catchment are summarised in Table S2. No evaporitic lithologies are reported in the catchment of Gaoping River in SW Taiwan based on 1:50,000 local geologic maps (Chen et al., 2001; Chen and Hsieh, 2016; Lin et al., 2011; Lin and Hong, 2012; Lin, 2013; Sung and Lin, 1993; Sung et al., 2000) and the 1:500,000 geologic map of Taiwan (Chen, 2000).

Table S2: Lithologies in the Gaoping River catchment

| Name (Epoch) | Description |
| --- | --- |
| Toukoshan Conglomerate  (Pleistocene) | Conglomerate with mudstone interbeds, intercalated with sheet or lenticular sandstone. Also known as Linkou Conglomerate. |
| Kueichiulin Formation  (Pleistocene -Pliocene) | Also known as Tashe Formation and Tangenshan sandstone. The former is mainly composed of thick mudstone with fine to coarse-grained sandstones and conglomerate interbeds. The latter is thick-bedded muddy sandstone, intercalated with alternated sandstone and shale. |
| Nanchuang Formation  (Pliocene -Miocene) | Thick beds of sandstone alternating with thin beds of shale. Also known as Wushan Formation. |
| Nankang Formation  (Miocene) | Alternating shale and sandstone, occasionally intercalated with thick beds of shale and sandstone. Also known as Changchihkeng Formation. |
| Lushan Formation  (Miocene) | Mainly composed of slate, argillite, alternations of metasandstone and slate and thick-bedded metasandstone. Also known as Chaochou Formation. |
| Chiayang Formation  (Eocene) | Slate with few thin-bedded metamorphosed sandstone. |
| Tachien Sandstone  (Eocene) | Thick-bedded, coarse grained to fine pebbly quartzose (meta-) sandstone, intercalated with argillite and slate. |
| Shihpachungchi Formation  (Eocene) | Thin alternations of slate and thin-bedded metamorphosed sandstone or siltstone, or diabase dike. |
| Pilushan Formation  (Eocene) | Slate, argillite, and metasandstone, occasionally intercalated with thin limestone. Also known as Gulo Formation. |
| Mesozoic black schist | Mainly black schist, green schist with few metachert. |

Ion chemistry

Water pH and conductivity was measured in the laboratory using a WTW pH-electrode SenTix 21 and WTW TetraCon325 conductivity probe. Ionic concentrations were measured on Dionex DX-120 ion chromatographs equipped with conductivity detectors. For separating and measuring cation concentrations, an isocratic mobile phase of 17 mM methanesulfonic acid at a flow rate of 1.3 ml/min on a Dionex IonPac CS12A 4x250 mm column with 25 µl sample injection volume was used. For separating and measuring anion concentrations, an isocratic mobile phase containing 2.7 mM NaHCO_3_ and 0.3 mM Na_2_CO_3_ at a flow rate of 1.3 ml/min on a Dionex IonPac AS12A 4x250 mm column with 25 µl sample injection volume was used. Blanks and in-house standards were measured to determine ion retention times and for calibration of detector response.

Constrained linear least-squares approach

The Matlab solver (lsqlin) for constrained linear-squares was used to deconvolve the contributions of carbonic acid weathering of silicate ($\alpha_{Silicate,H_{2}CO_{3}}$) and carbonate ($\alpha_{Carbonate,H_{2}CO_{3}}$) and sulphuric acid weathering of silicate ($\alpha_{Silicate,H_{2}SO_{4}}$) and carbonate ($\alpha_{Carbonate,H_{2}SO_{4}}$). Their relative contributions are expressed in the vector $\bar{\alpha}$.

$$\bar{\alpha}=\left( \begin{matrix} \alpha_{Silicate,H_{2}CO_{3}} \\ \alpha_{Silicate,H_{2}SO_{4}} \\ \alpha_{Carbonate,H_{2}CO_{3}} \\ \alpha_{Carbonate,H_{2}SO_{4}} \end{matrix} \right)$$

The elements of vector $\bar{\alpha}$ are constrained by being equal to or greater than 0 and less than or equal to 1, corresponding to 0 and 100%, respectively.

$$\left( \begin{matrix} 0 \\ 0 \\ 0 \\ 0 \end{matrix} \right)\leq\bar{\alpha}\leq\left( \begin{matrix} 1 \\ 1 \\ 1 \\ 1 \end{matrix} \right)$$

Three sets of linear equality constraints are imposed on the model output $\bar{\alpha}$: (1) the sum of the elements in $\bar{\alpha}$ must equal 1, (2) measured and modelled mineral unit-normalised sulphate concentrations (SO4_m_) must be equal so that the model output conforms to the quaternary model mixing line, and (3) modelled and measured radiocarbon isotopic composition of dissolved inorganic carbon must be equal. Here, the measured radiocarbon isotopic composition of dissolved inorganic carbon is given as PMC_m_. This is related to PMC_modern_, which is the radiocarbon isotopic composition of the carbonic acid involved in the weathering reactions. These equality constraints are formally expressed by:

$$beq=Aeq\times\bar{\alpha}$$

$$\left( \begin{matrix} 1 \\ {SO4}_{m} \\ 0 \end{matrix} \right)=\left( \begin{matrix} 1 & 1 & 1 & 1 \\ 0 & 1 & 0 & 0.5 \\ 2\times\left( {PMC}_{modern}-{PMC}_{m} \right) & 0 & \left( {PMC}_{modern}-2\times{PMC}_{m} \right) & -{PMC}_{m} \end{matrix} \right)\times\bar{\alpha}$$

The solution space spanned by $\bar{\alpha}$ with the imposed constraints, is solved following the least-squares method using the equations:

$$d=C\times\bar{\alpha}$$

$$\left( \begin{matrix} Ca{SO}_{4}HCO_{3} \\ Mg{SO}_{4}HCO_{3} \\ Na{SO}_{4}HCO_{3} \end{matrix} \right)=\left( \begin{matrix} {ws}/2 & ws & {wc}/2 & {wc}/{1.5} \\ {xs}/2 & xs & {xc}/2 & {xc}/{1.5} \\ {ys}/2 & ys & {yc}/2 & {yc}/{1.5} \end{matrix} \right)\times\bar{\alpha}$$

On the left-hand side of this equation, from top to bottom is the molar ratio of calcium, magnesium, and sodium normalised to the molar sum of sulphate and bicarbonate, corresponding to $Ca{SO}_{4}HCO_{3}$, $Mg{SO}_{4}HCO_{3}$, and $Na{SO}_{4}HCO_{3}$, respectively. These are related in a linearised fashion to the relative abundance of calcium released by silicates (ws) and carbonates (wc), magnesium by silicates (xs) and carbonates (xc), and sodium by silicates (ys) and carbonates (yc) normalised to the sum of anions released by the four weathering reaction pathways. The relative abundance of the cations are calculated using the ideal mineral units of (Ca,Mg,2Na,2K)SiO_3_ for silicates and (Ca,Mg,2Na,2K)CO_3_ for carbonates. In both silicates and carbonates, the charge balance of the summed cations must equal 2. Mineral formulas are generated to test model output uncertainty as a function of uncertainty in the chemistry of weathered mineral species modified after Gaillardet et al. (1999) and Burke et al. (2018). The calculated stoichiometries for silicates and carbonates are then normalised to the molar sum of major anions (sulphate and bicarbonate) generated for each mineral unit weathered for each reaction pathway as given in Table 1.

Table S3: Silicate and carbonate major chemistry used to generate the ideal mineral unit formula and the major anion amount generated from each reaction pathway.

|  | Ca/Na (µ±σ) | Mg/Na (µ±σ) | K/Na (µ±σ) | HCO_3_+SO_4_ |
| --- | --- | --- | --- | --- |
| $\alpha_{Silicate,H_{2}CO_{3}}$ | 0.35±0.125 | 0.25±0.1 | 0.16±0.032 | 2 |
| $\alpha_{Silicate,H_{2}SO_{4}}$ |  |  |  | 1 |
| $\alpha_{Carbonate,H_{2}CO_{3}}$ | 60±15 | 30±7.5 | 0.5±0.1 | 2 |
| $\alpha_{Carbonate,H_{2}SO_{4}}$ |  |  |  | 1.5 |


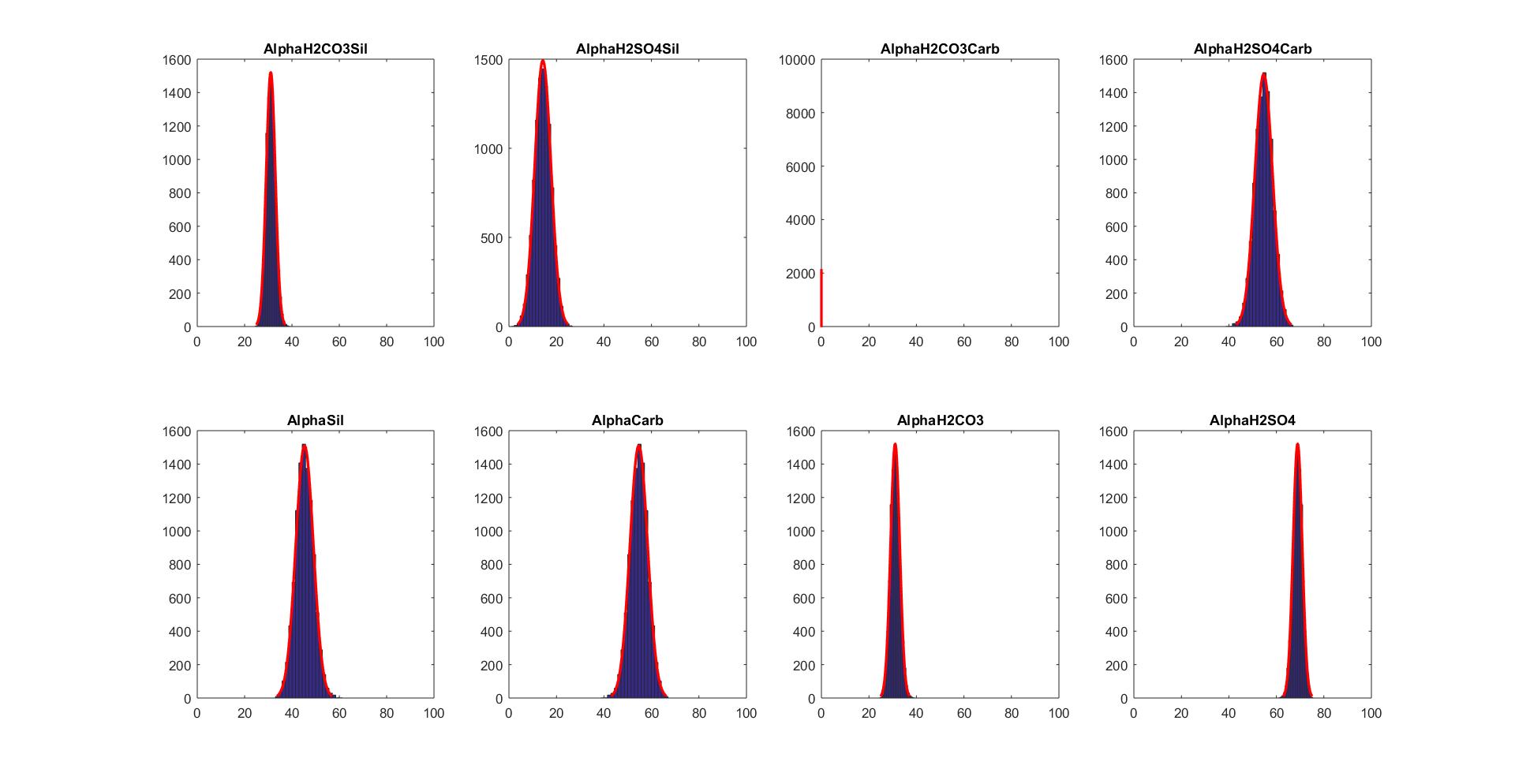


Figure S1: Model output for the Ailiao River (2017JuneSDM) with 10000 simulations displaying model uncertainty. From top left to top right percent: contributions from $\alpha_{Silicate,H_{2}CO_{3}}$, $\alpha_{Silicate,H_{2}SO_{4}}$, $\alpha_{Carbonate,H_{2}CO_{3}}$, and $\alpha_{Carbonate,H_{2}SO_{4}}$. From bottom left to bottom right: summed contributions from silicate, carbonate, carbonic acid, and sulfuric acid weathering.


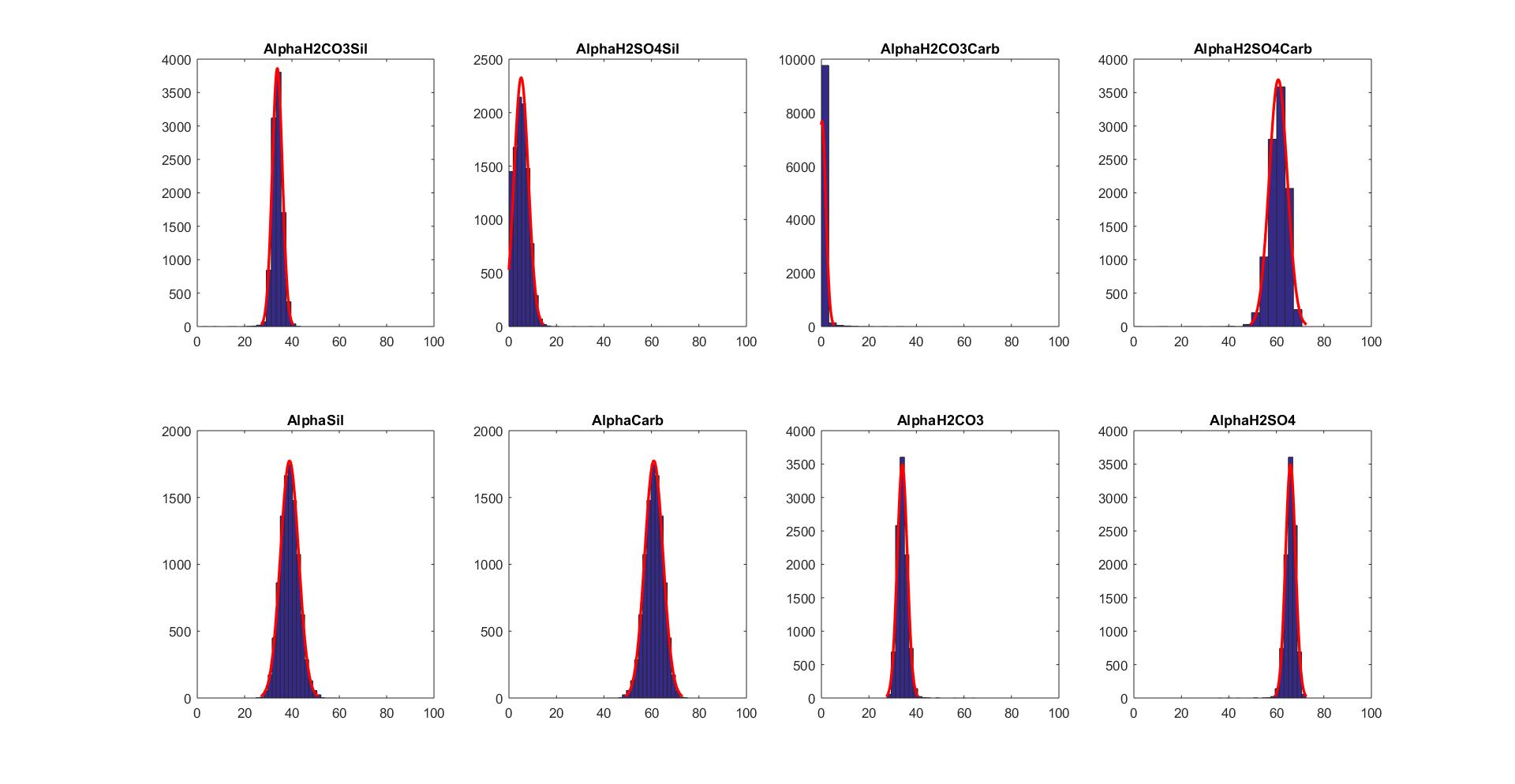


Figure S2: Model output for the Laonong River (2017JuneLR) with 10000 simulations displaying model uncertainty. From top left to top right percent: contributions from $\alpha_{Silicate,H_{2}CO_{3}}$, $\alpha_{Silicate,H_{2}SO_{4}}$, $\alpha_{Carbonate,H_{2}CO_{3}}$, and $\alpha_{Carbonate,H_{2}SO_{4}}$. From bottom left to bottom right: summed contributions from silicate, carbonate, carbonic acid, and sulfuric acid weathering.


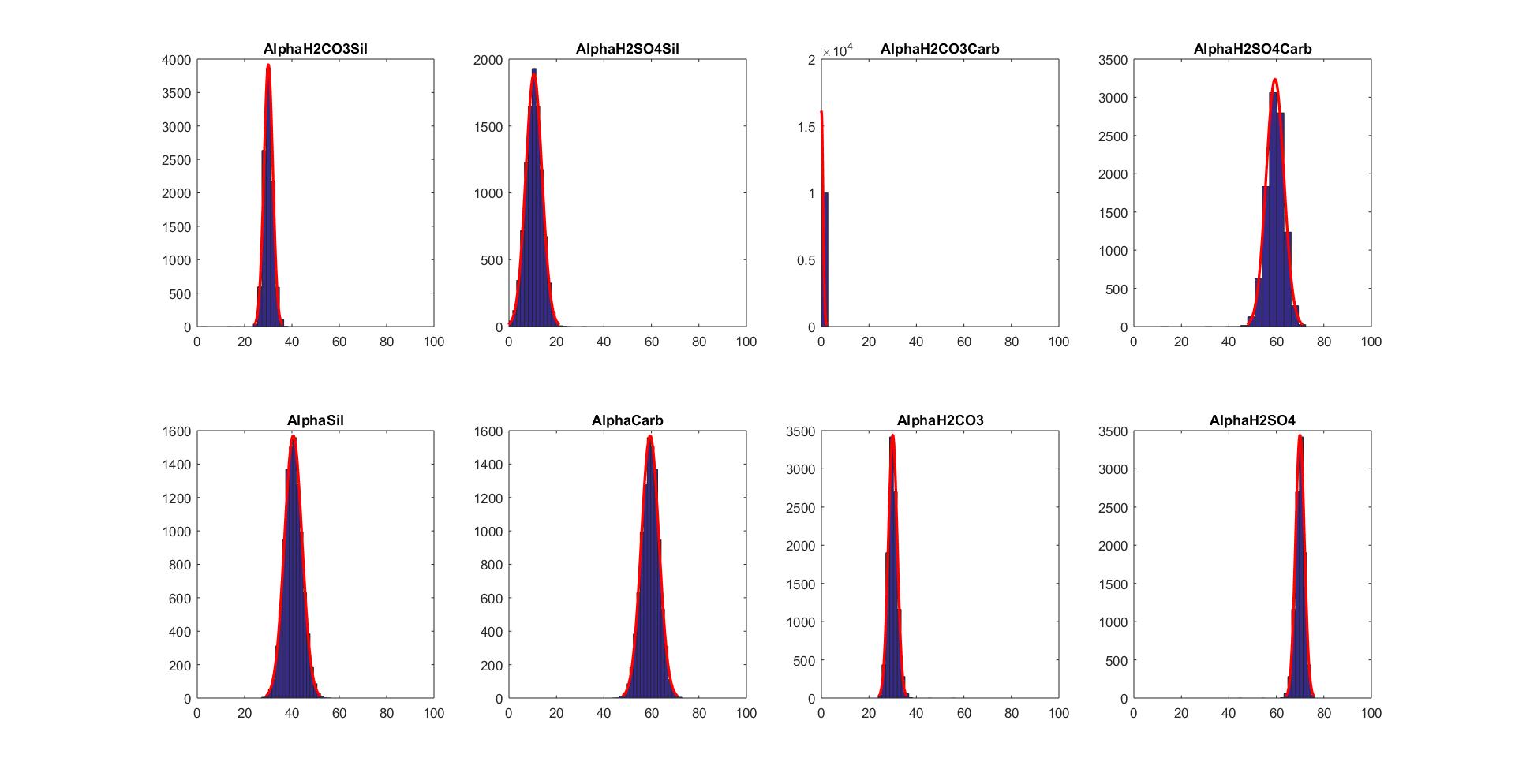


Figure S3: Model output for the Ailiao River (2017JuneAR) with 10000 simulations displaying model uncertainty. From top left to top right percent: contributions from $\alpha_{Silicate,H_{2}CO_{3}}$, $\alpha_{Silicate,H_{2}SO_{4}}$, $\alpha_{Carbonate,H_{2}CO_{3}}$, and $\alpha_{Carbonate,H_{2}SO_{4}}$. From bottom left to bottom right: summed contributions from silicate, carbonate, carbonic acid, and sulfuric acid weathering.


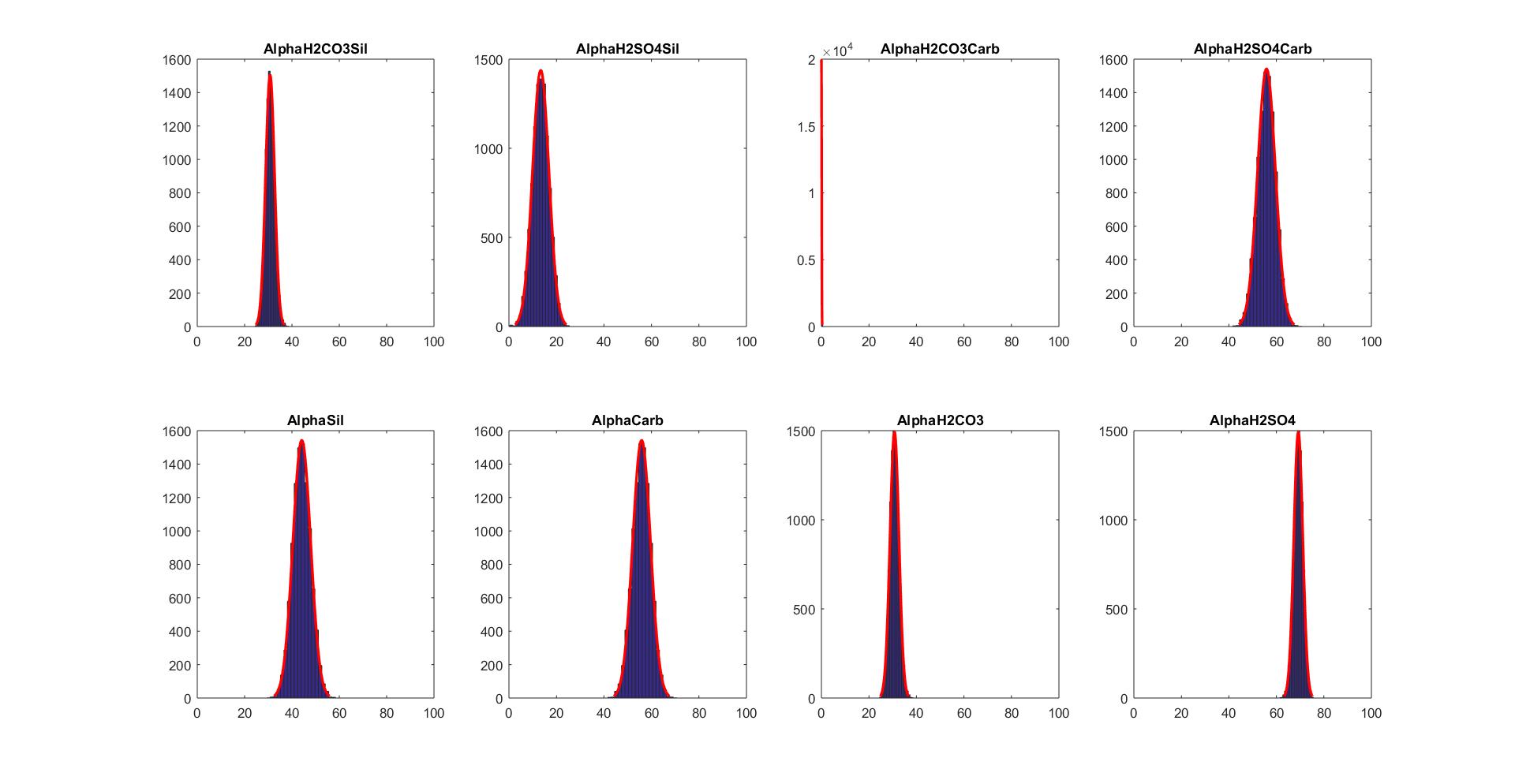


Figure S4: Model output for the Laonong River (2017June181) with 10000 simulations displaying model uncertainty. From top left to top right percent: contributions from $\alpha_{Silicate,H_{2}CO_{3}}$, $\alpha_{Silicate,H_{2}SO_{4}}$, $\alpha_{Carbonate,H_{2}CO_{3}}$, and $\alpha_{Carbonate,H_{2}SO_{4}}$. From bottom left to bottom right: summed contributions from silicate, carbonate, carbonic acid, and sulfuric acid weathering.


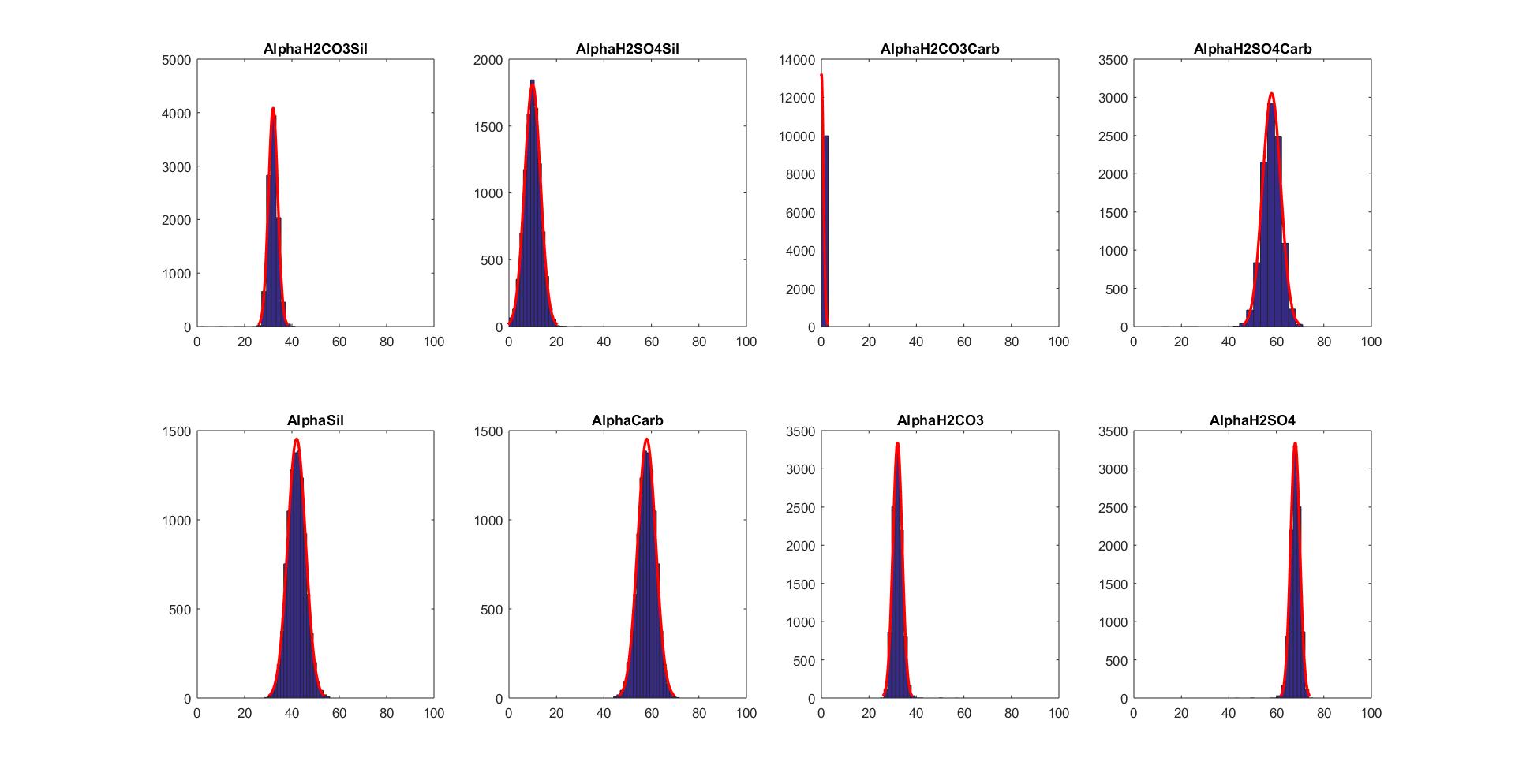


Figure S5: Model output for the Laonong River (2017OctLR) with 10000 simulations displaying model uncertainty. From top left to top right percent: contributions from $\alpha_{Silicate,H_{2}CO_{3}}$, $\alpha_{Silicate,H_{2}SO_{4}}$, $\alpha_{Carbonate,H_{2}CO_{3}}$, and $\alpha_{Carbonate,H_{2}SO_{4}}$. From bottom left to bottom right: summed contributions from silicate, carbonate, carbonic acid, and sulfuric acid weathering.


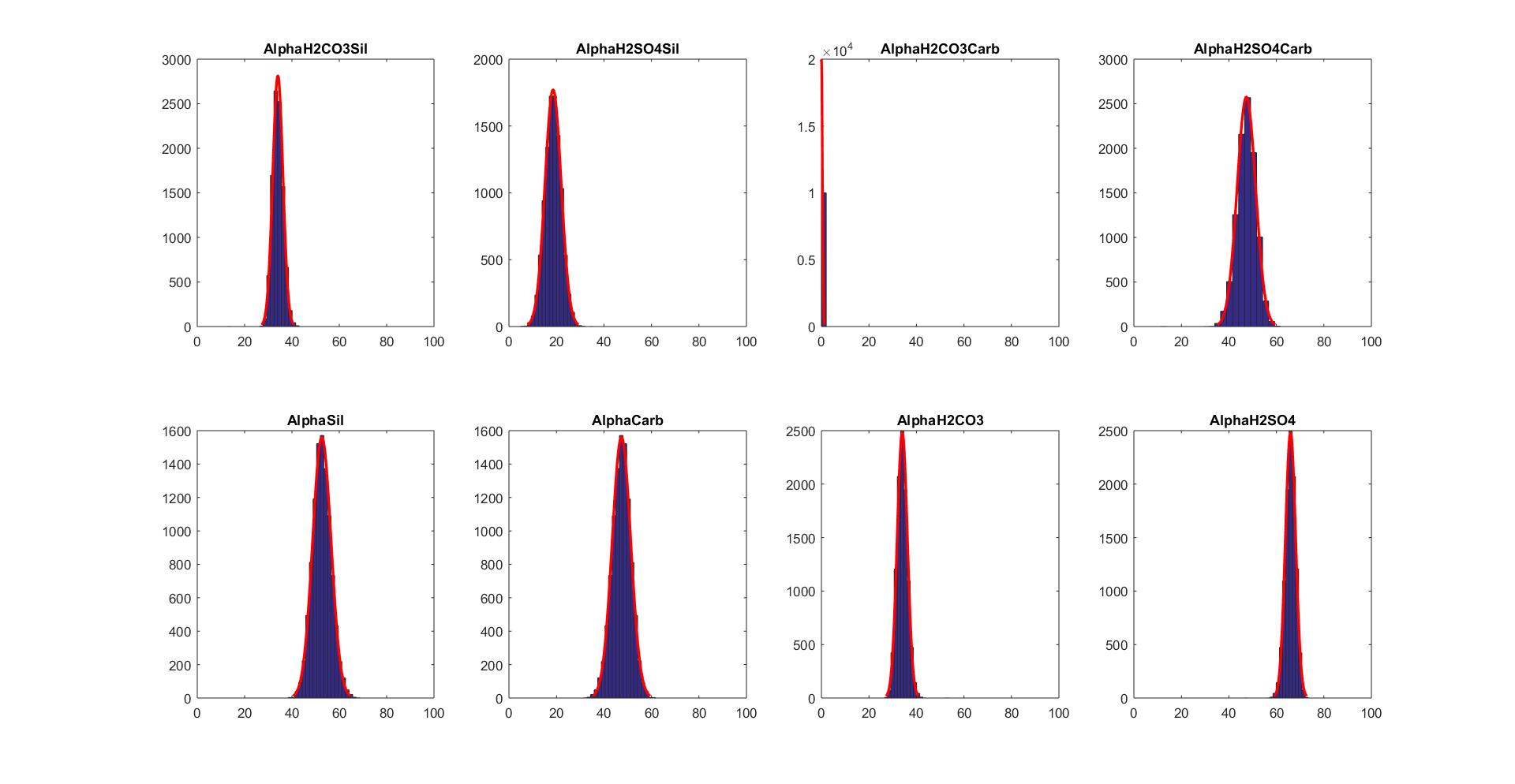


Figure S6: Model output for the Laonong River (2017Oct181) with 10000 simulations displaying model uncertainty. From top left to top right percent: contributions from $\alpha_{Silicate,H_{2}CO_{3}}$, $\alpha_{Silicate,H_{2}SO_{4}}$, $\alpha_{Carbonate,H_{2}CO_{3}}$, and $\alpha_{Carbonate,H_{2}SO_{4}}$. From bottom left to bottom right: summed contributions from silicate, carbonate, carbonic acid, and sulfuric acid weathering.


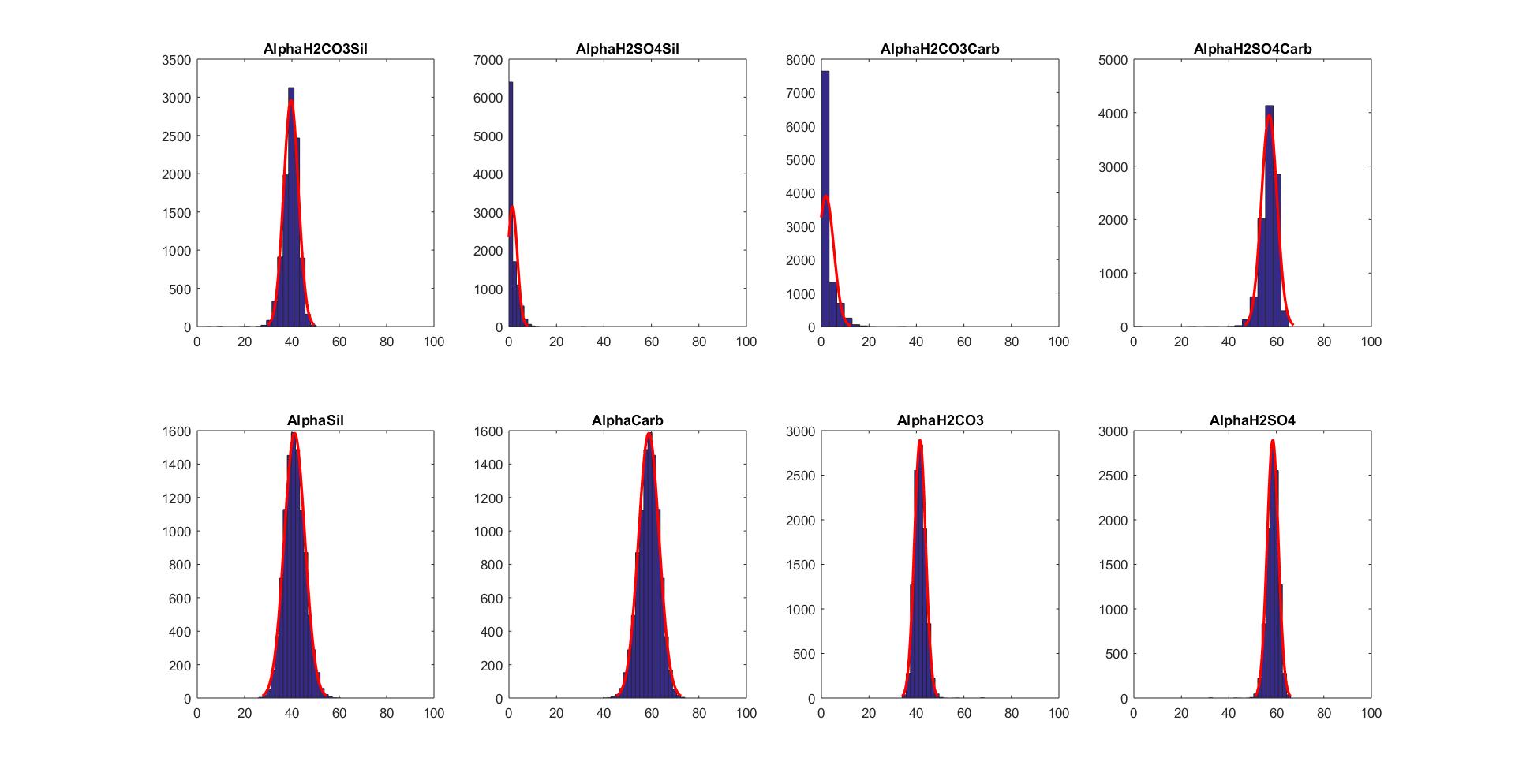


Figure S7: Model output for the Ailiao River (2017OctAR) with 10000 simulations displaying model uncertainty. From top left to top right percent: contributions from $\alpha_{Silicate,H_{2}CO_{3}}$, $\alpha_{Silicate,H_{2}SO_{4}}$, $\alpha_{Carbonate,H_{2}CO_{3}}$, and $\alpha_{Carbonate,H_{2}SO_{4}}$. From bottom left to bottom right: summed contributions from silicate, carbonate, carbonic acid, and sulfuric acid weathering.


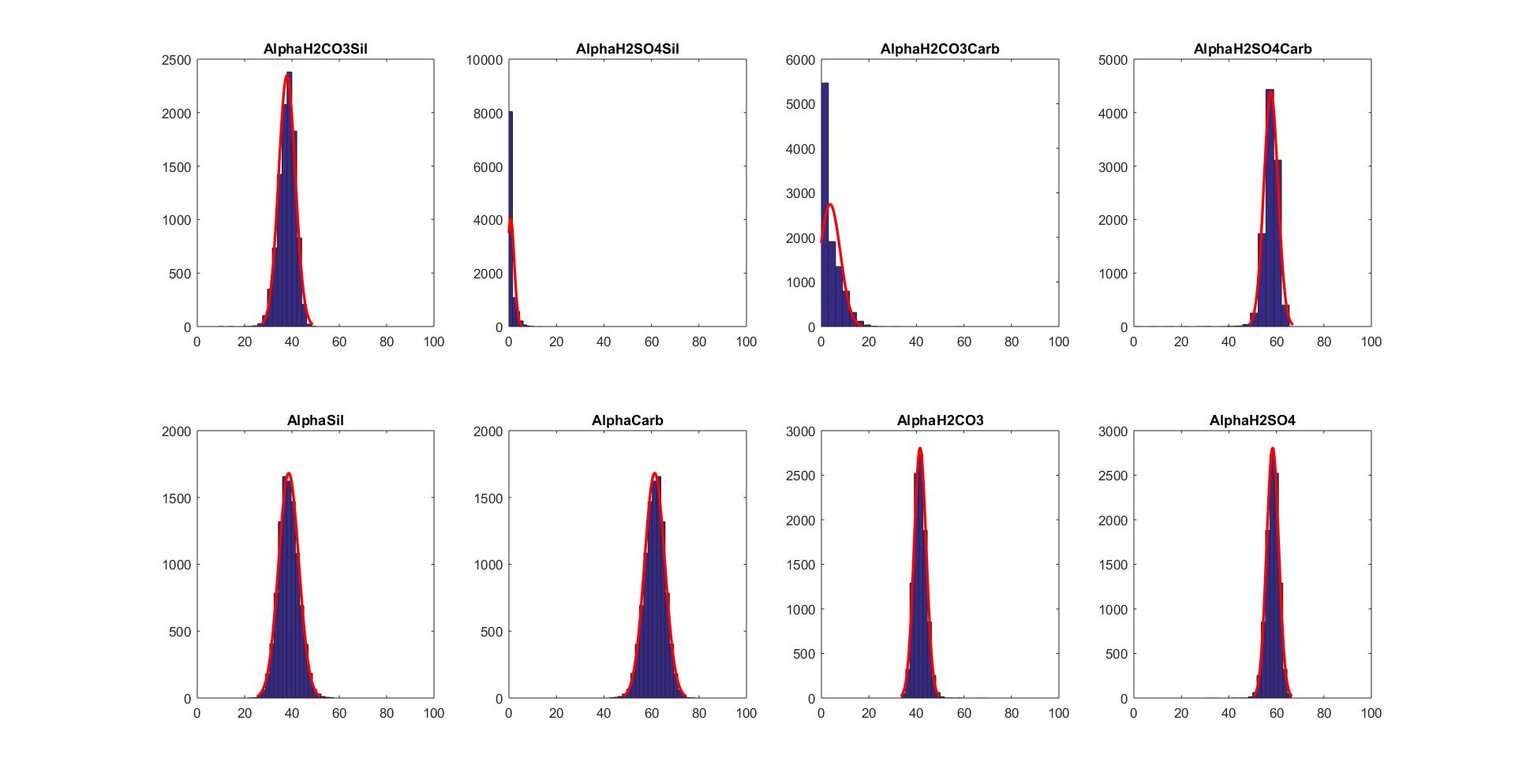


Figure S8: Model output for the Ailiao River (2017OctSDM) with 10000 simulations displaying model uncertainty. From top left to top right percent: contributions from $\alpha_{Silicate,H_{2}CO_{3}}$, $\alpha_{Silicate,H_{2}SO_{4}}$, $\alpha_{Carbonate,H_{2}CO_{3}}$, and $\alpha_{Carbonate,H_{2}SO_{4}}$. From bottom left to bottom right: summed contributions from silicate, carbonate, carbonic acid, and sulfuric acid weathering.


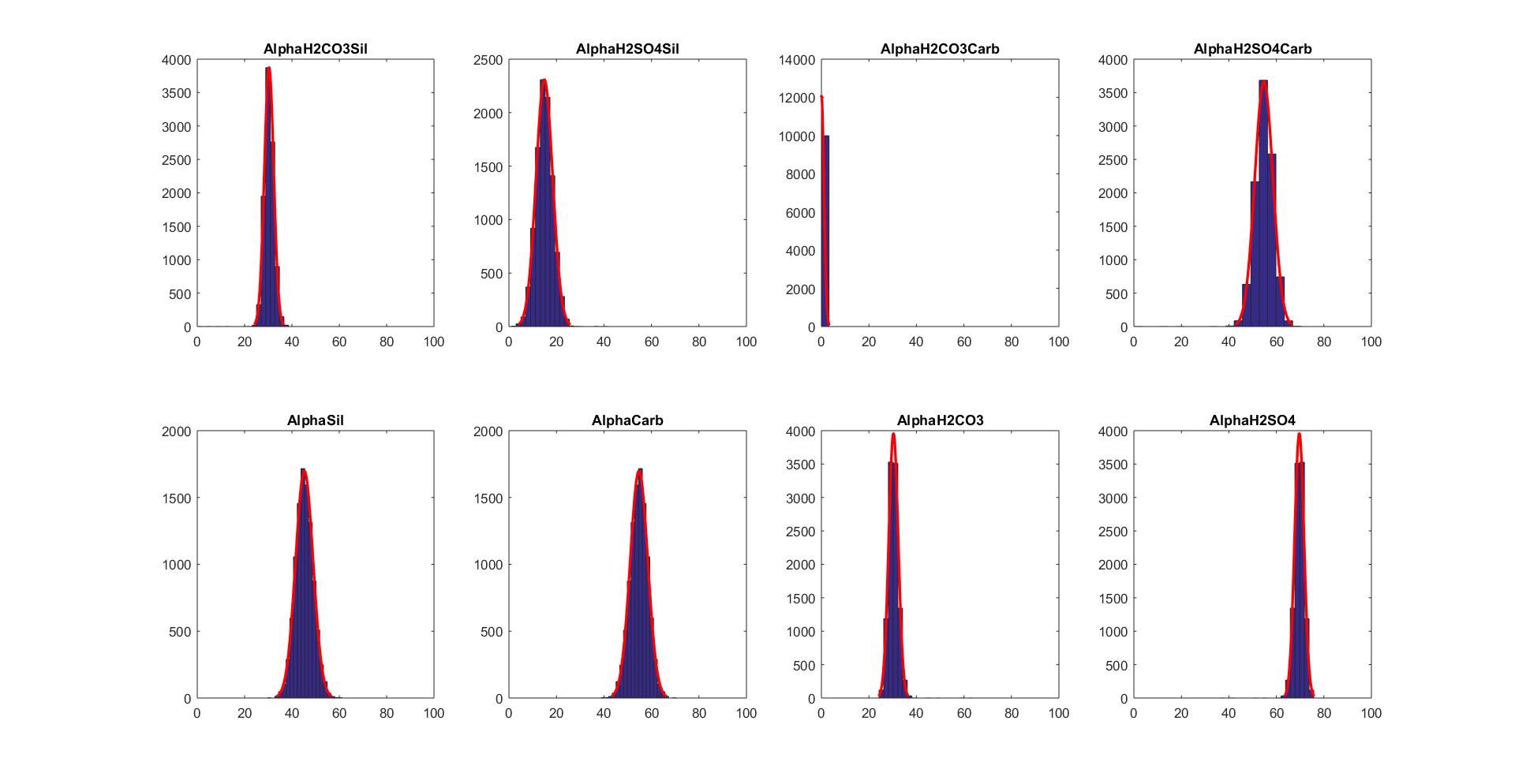


Figure S9: Model output for the Laonong River (2018FebLR) with 10000 simulations displaying model uncertainty. From top left to top right percent: contributions from $\alpha_{Silicate,H_{2}CO_{3}}$, $\alpha_{Silicate,H_{2}SO_{4}}$, $\alpha_{Carbonate,H_{2}CO_{3}}$, and $\alpha_{Carbonate,H_{2}SO_{4}}$. From bottom left to bottom right: summed contributions from silicate, carbonate, carbonic acid, and sulfuric acid weathering.


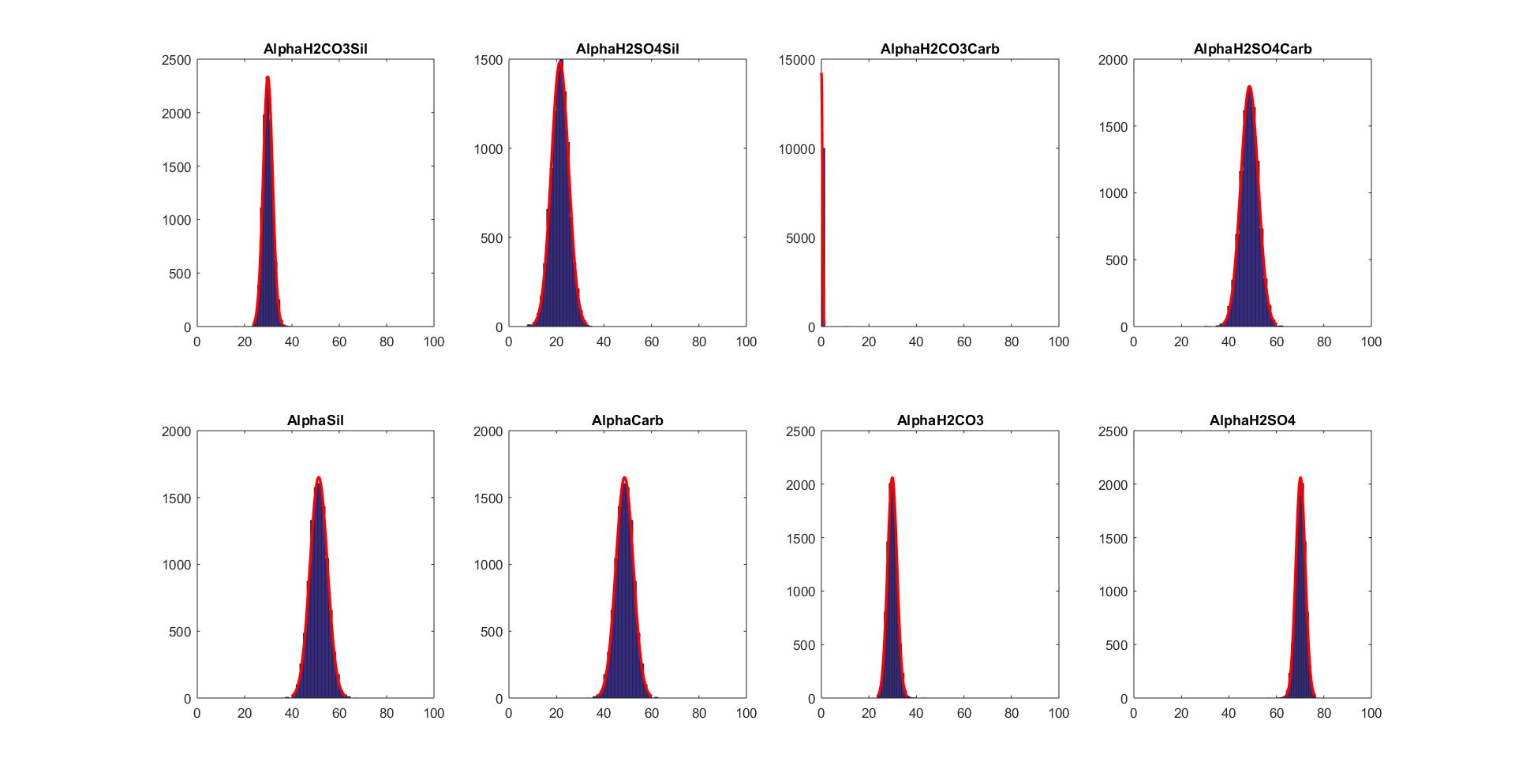


Figure S10: Model output for the Laonong River (2018Feb181) with 10000 simulations displaying model uncertainty. From top left to top right percent: contributions from $\alpha_{Silicate,H_{2}CO_{3}}$, $\alpha_{Silicate,H_{2}SO_{4}}$, $\alpha_{Carbonate,H_{2}CO_{3}}$, and $\alpha_{Carbonate,H_{2}SO_{4}}$. From bottom left to bottom right: summed contributions from silicate, carbonate, carbonic acid, and sulfuric acid weathering.


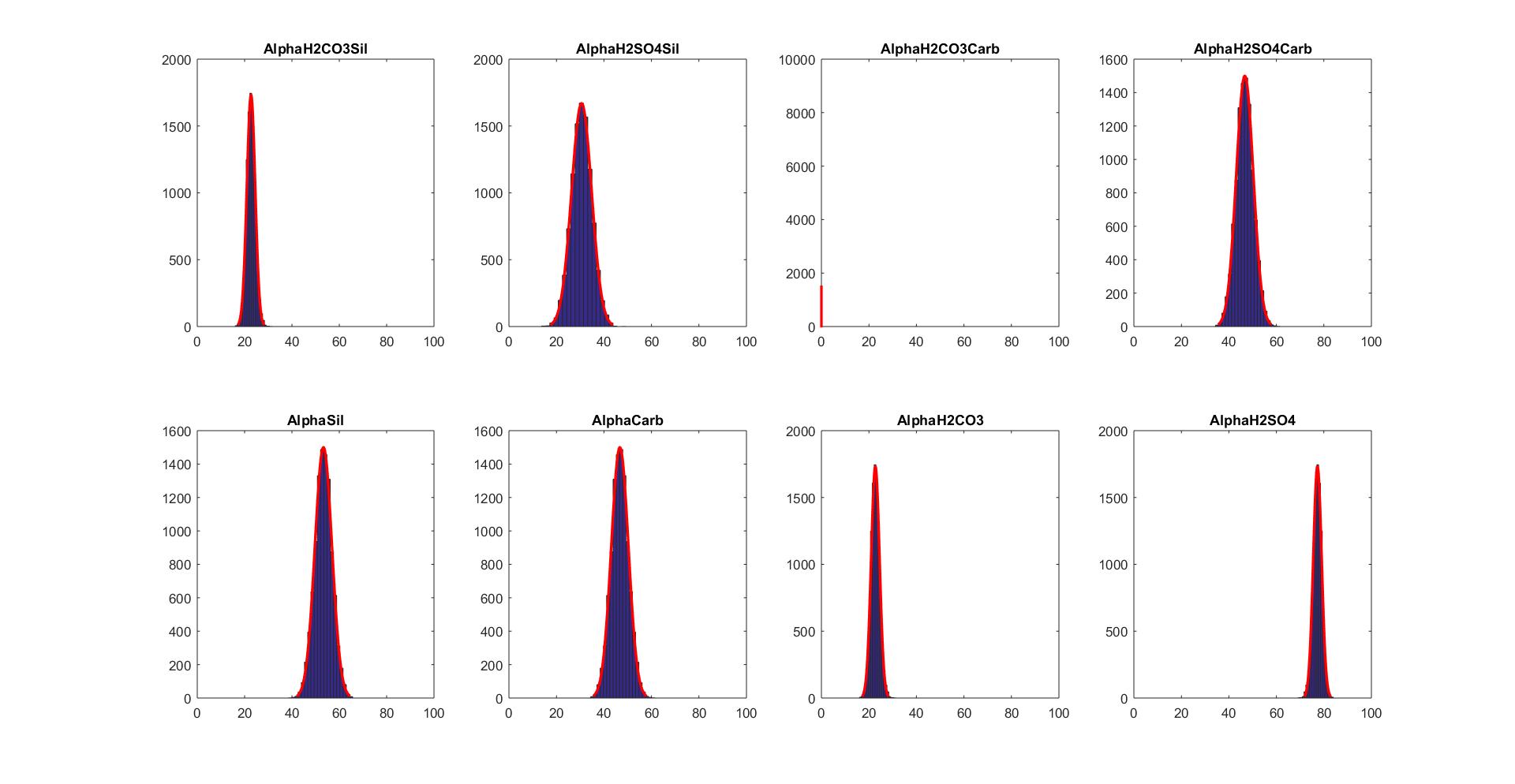


Figure S11: Model output for the Ailiao River (2018FebSDM) with 10000 simulations displaying model uncertainty. From top left to top right percent: contributions from $\alpha_{Silicate,H_{2}CO_{3}}$, $\alpha_{Silicate,H_{2}SO_{4}}$, $\alpha_{Carbonate,H_{2}CO_{3}}$, and $\alpha_{Carbonate,H_{2}SO_{4}}$. From bottom left to bottom right: summed contributions from silicate, carbonate, carbonic acid, and sulfuric acid weathering.


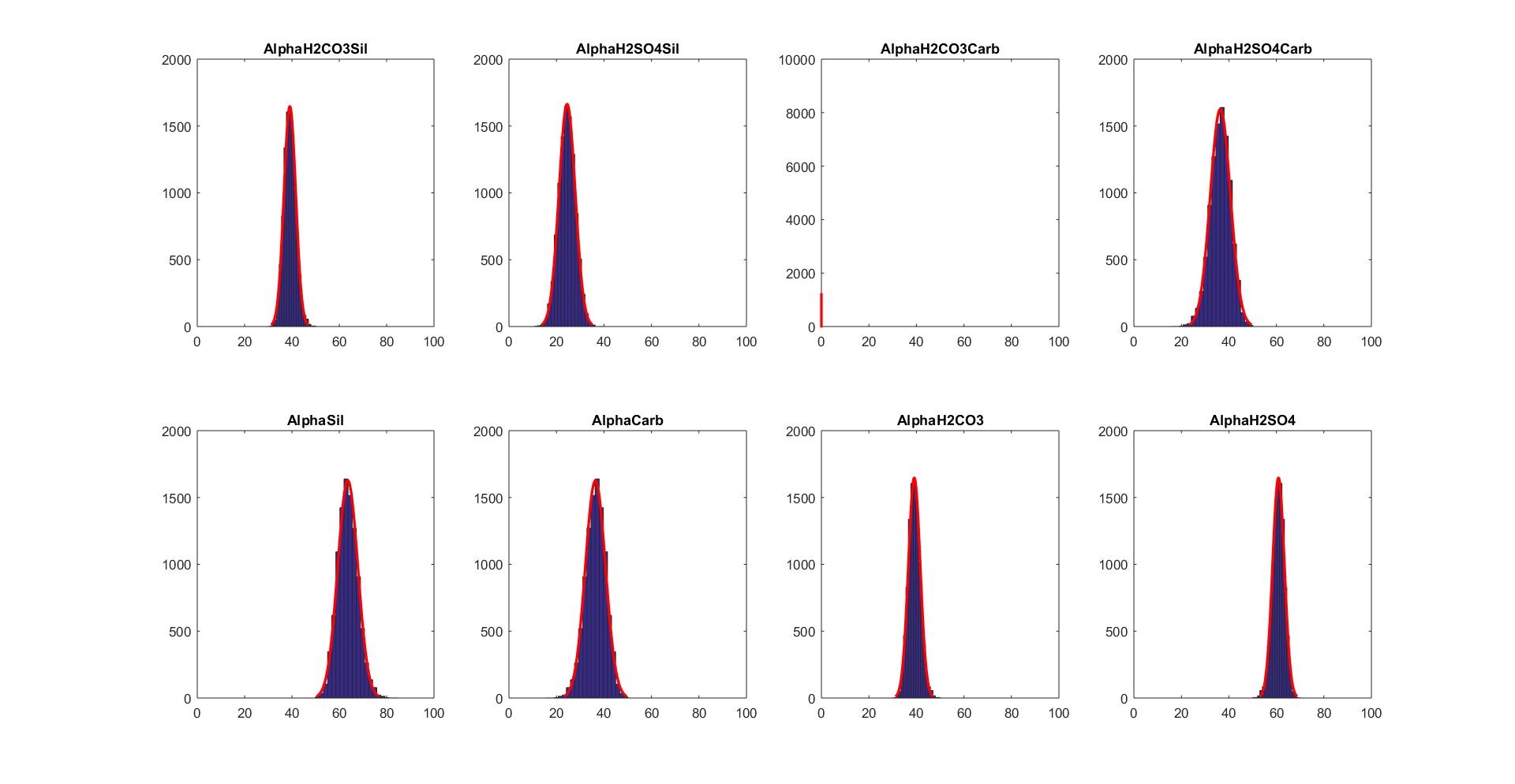


Figure S12: Model output for the mainstem of the Gaoping River (2018FebLG) with 10000 simulations displaying model uncertainty. From top left to top right percent: contributions from $\alpha_{Silicate,H_{2}CO_{3}}$, $\alpha_{Silicate,H_{2}SO_{4}}$, $\alpha_{Carbonate,H_{2}CO_{3}}$, and $\alpha_{Carbonate,H_{2}SO_{4}}$. From bottom left to bottom right: summed contributions from silicate, carbonate, carbonic acid, and sulfuric acid weathering.


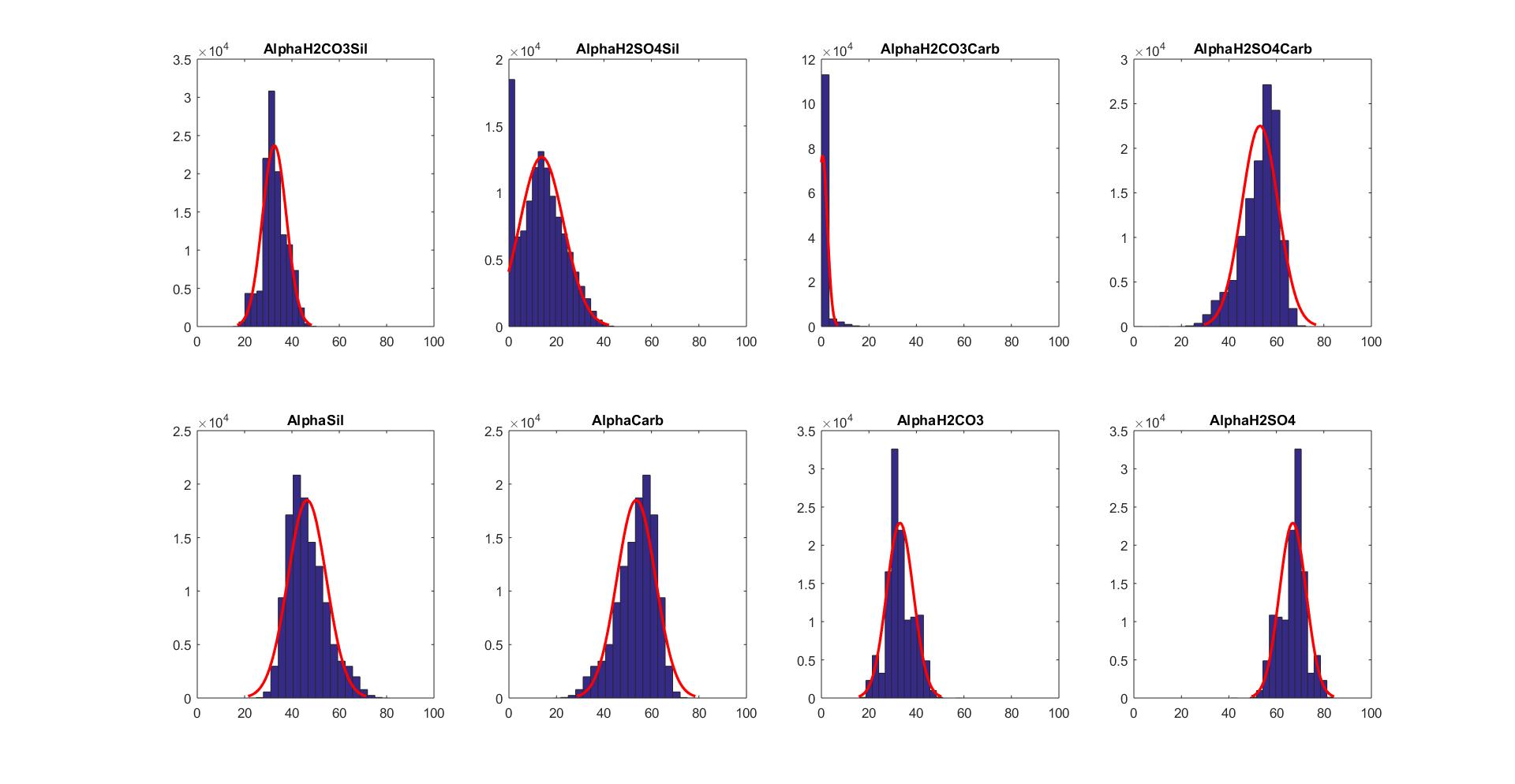


Figure S13: Model output for summed 12 Gaoping River samples with 10000 simulations displaying model uncertainty. From top left to top right percent: contributions from $\alpha_{Silicate,H_{2}CO_{3}}$, $\alpha_{Silicate,H_{2}SO_{4}}$, $\alpha_{Carbonate,H_{2}CO_{3}}$, and $\alpha_{Carbonate,H_{2}SO_{4}}$. From bottom left to bottom right: summed contributions from silicate, carbonate, carbonic acid, and sulfuric acid weathering.


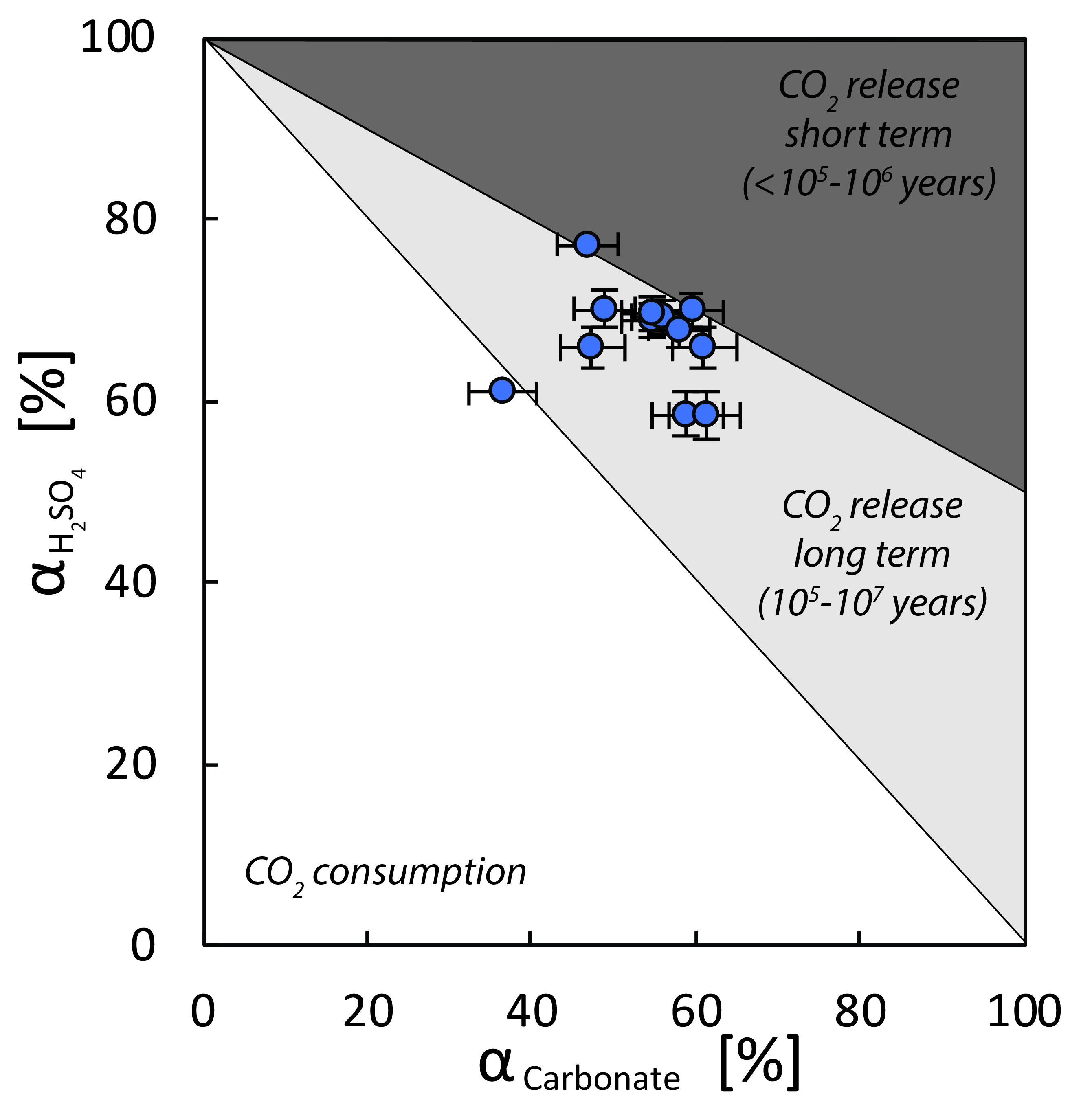


Figure S14: Figure emulated after Torres et al. (2016; Fig. 7) with data generated in this study. The errors show 1-σ uncertainty as calculated using the Monte Carlo approach. The interpretations made in this study are consistent with the framework proposed by Torres et al. (2016), which combines arguments of alkalinity and DIC concentrations released to the ocean as a result of chemical weathering, with Taiwan releasing CO_2_ over geological time scales.

**Comparisons to other Taiwanese rivers**

Figure S15: Sulphate versus bicarbonate mineral-unit normalised concentrations corrected for cyclic salt input using methods described. The observations are in concordance with simple carbonic and sulphuric acid weathering of silicate and carbonate minerals, with sulphuric acid weathering of silicates exhibiting an endmember of (0,1), sulphuric acid weathering of carbonates exhibiting an end member of (1,0.5), and carbonic acid weathering of silicates and carbonates both sharing the same end member at (2,0). Contributions of evaporitic minerals such as halite or sylvite, would divert the dissolved ion chemistry in the direction of the origin of the plot. The data shown here includes 182 observations from the Liwu River (Calmels et al., 2011) and 107 observations from the Tamaili and Chenyoulan drainage basins (Emberson et al., accepted). The Emberson et al. (accepted) dataset include 2 anomalous observations (one from a hot spring, and one from a landslide seepage), which calculated negative values, which could not be shown here.

The relationship of mineral unit-normalised sulphate to bicarbonate concentrations in the Liwu, Chenyoulan, and Taimali Rivers illustrate that similar weathering patterns are active with those of the Gaoping River. Values of <1 HCO_3_ and >0.5 SO_4_ mineral unit normalised concentrations show that sulfuric acid weathering of silicates must be operating in this catchment as well. Additionally, the many observations centred around ~1 HCO_3_ and ~0.5 SO_4_ suggest that sulfuric acid weathering of carbonate is likely important. The overwhelming number of observations from Taiwanese rivers adhere to the quaternary weathering mixing line that can be theoretically expected from silicate and carbonate mineral weathering. For the Liwu River data from Calmels et al. (2011), the observations during Typhoon and under normal conditions shows all data points adhering to the quaternary mixing line (R^2^=1.00; y=-0.52x+1.02) also indicate that seasonal and hydrological variations do not induce divergence from this simple mineral weathering behaviour. Albeit DI^14^C data is unavailable for these river systems, the dissolved ion patterns are consistent with similar chemical weathering behaviour. Thus, supporting the extrapolation of findings from the Gaoping River to the entire island of Taiwan.

**Other Weathering Plots**

Figure S16: Mixing diagram sensu Gaillardet et al. 1999 showing the contributions of silicate and carbonate weathering to dissolved ion load in the Gaoping River. Concentrations given as molar ratios without correction for cyclic inputs.

Figure S16 shows the mixing between silicate and carbonate end members based their typical endmember ranges. The relative contributions of silicate and carbonate-derived ion composition indicated by the Gaillardet plot are consistent with the results generated using the new model as shown in figure S13.

**References**

Burke, A., Present, T. M., Paris, G., Rae, E. C. M., Sandilands, B. H., Gaillardet, J., Peucker-Ehrenbrink, B., Fischer, W. W., McClelland, J. W., Spencer, R. G. M., Voss, B. M., and Adkins, J. F., 2018, Sulfur isotopes in rivers: Insights into global weathering budgets, pyrite oxidation, and the modern sulfur cycle: Earth and Planetary Science Letters, v. 496, p. 168-177.

Calmels, D., Galy, A., Hovius, N., Bickle, M., West, A. J., Chen, M.-C., and Chapman, H., 2011, Contribution of deep groundwater to the weathering budget in a rapidly eroding mountain belt, Taiwan: Earth and Planetary Science Letters, v. 303, no. 1, p. 48-58.

Chen, Chao-Hsia, 2000, 1:500,000 Geologic Map of Taiwan. Central Geological Survey, Ministry of Economic Affairs, Taiwan R.O.C.

Chen, Hua-Wen, Wu, Leh-Chyun, Shea, Kai-Shuan, and Ho, Hsin-Chang, 2001, Geological map of Taiwan, Scale 1:50,000, Sheet 61, Kaohsiung. Central Geological Survey, Ministry of Economic Affairs, Taiwan R.O.C.

Chen, Mien-Ming and Hsieh, Yu-Chung, 2016, Geological map of Taiwan, Scale 1:50,000, Sheet 39, Alishan. Central Geological Survey, Ministry of Economic Affairs, Taiwan R.O.C.

Chung, C.-H., You, C.-F. and Chu, H.-Y. (2009) Weathering sources in the Gaoping (Kaoping) river catchments, southwestern Taiwan: Insights from major elements, Sr isotopes, and rare earth elements. Journal of Marine Systems 76, 433-443.

Emberson, R., Hovius, N., Galy, A. and Odin, M. (2016) Oxidation of sulfides and rapid weathering in recent landslides. Earth Surface Dynamics 4, 727-742.

Gaillardet, J., Dupré, B., Louvat, P., and Allègre, C. J., 1999, Global silicate weathering and CO2 consumption rates deduced from the chemistry of large rivers: Chemical Geology, v. 159, no. 1, p. 3-30.

Lin, Chii-Wen, Lin, Wei-Hsiung, and Kao, Ming-Chien, 2011, Geological map of Taiwan, Scale 1:50,000, Sheet 62, Chaozhou. Central Geological Survey, Ministry of Economic Affairs, Taiwan R.O.C.

Lin, Chii-Wen, 2013, Geological map of Taiwan, Scale 1:50,000, Sheet 56, Qishan. Central Geological Survey, Ministry of Economic Affairs, Taiwan R.O.C.

Lin, Chii-Wen and Hong, Guo-Teng (2012) Geological map of Taiwan, Scale 1:50,000, Sheet 57, Meinong. Central Geological Survey, Ministry of Economic Affairs, Taiwan R.O.C.

Liu, Y.-C., You, C.-F., Huang, K.-F., Wang, R.-M., Chung, C.-H. and Liu, H.-C. (2012) Boron sources and transport mechanisms in river waters collected from southwestern Taiwan: Isotopic evidence. Journal of Asian Earth Sciences 58, 16-23.

Sung, Quocheng and Lin, Wei-Hsiung, 1993, Geological map of Taiwan, Scale 1:50,000, Sheet 67, Fangliao. Central Geological Survey, Ministry of Economic Affairs, Taiwan R.O.C.

Sung, Quocheng, Lin, Ching-Wei, Lin, Wei-Hsiung, and Lin, Wen-Cheng, 2000, Geological map of Taiwan scale 1:50,000, Sheet 51, Chiahsien. Central Geological Survey, Ministry of Economic Affairs, Taiwan R.O.C.
